# The Prognostic Value of Thrombocytopenia in COVID-19 Patients; a Systematic Review and Meta-Analysis

**Published:** 2020-09-19

**Authors:** Davood Bashash, Fatemeh Sadat Hosseini-Baharanchi, Mostafa Rezaie-Tavirani, Majid Safa, Nader Akbari Dilmaghani, Mohammad Faranoush, Hassan Abolghasemi

**Affiliations:** 1 Department of Hematology and Blood Banking, School of Allied Medical Sciences, Shahid Beheshti University of Medical Sciences, Tehran, Iran.; 2 Minimally Invasive Surgery Research Center & Department of Biostatistics, School of Public Health, Iran University of Medical Sciences, Tehran, Iran.; 3 Proteomics Research Center, Faculty of Paramedical Sciences, Shahid Beheshti University of Medical Sciences, Tehran, Iran.; 4 Department of Hematology and Blood Banking, Faculty of Allied Medicine, Iran University of Medical Sciences, Tehran, Iran.; 5 Hearing Disorders Research Center, Loghman Hakim Hospital, Shahid Beheshti University of Medical Sciences, Tehran, Iran.; 6 Department of Otolaryngology, Head and Neck Surgery, Loghman Hakim Educational Hospital, School of Medicine, Shahid Beheshti University of Medical Sciences, Tehran, Iran.; 7 Pediatric Growth and Development Research Center, Institute of Endocrinology, Iran University of Medical Sciences, Tehran, Iran.; 8 Pediatric Congenital Hematologic Disorders Research Center, Shahid Beheshti University of Medical Sciences, Tehran, Iran.

**Keywords:** SARS-CoV-2, COVID-19, Prognosis, Blood Platelets, Thrombocytopenia, Meta-analysis

## Abstract

**Introduction:**

Multiple lines of evidence have attested that decreased numbers of platelets may serve as a surrogate marker for poor prognosis in a wide range of infectious diseases. Thus, to provide a well-conceptualized viewpoint demonstrating the prognostic value of thrombocytopenia in COVID-19, we performed a meta-analysis of pertinent literature.

**Methods:**

The keywords “platelet” OR “thrombocytopenia” AND “COVID-19” OR “coronavirus 2019” OR “2019-nCoV” OR “SARS-CoV-2” were searched in National Library of Medicine Medline/PubMed and Scopus between December 30, 2019, and May 9, 2020 in English without any restriction. The initial search results were first screened by title and abstract, and then full texts of relevant articles representing information on the platelet count (main outcome) with a clinically validated deﬁnition of COVID-19 severity were ﬁnally selected. To assess the existence of bias in the included studies, the funnel plot and egger plot along with egger tests were used. Also, the heterogeneity among the included studies was tested using the Chi-square test.

**Results:**

The results of our meta-analysis of 19 studies, totaling 3383 COVID-19 patients with 744 (21.9%) severe cases, revealed that non-severe cases have a significantly higher number of platelets and showed that the probability of the emergence of thrombocytopenia is significantly higher in the severe cases with the pooled mean difference of -21.5 (%95 CI: -31.57, -11.43).

**Conclusion:**

Decreased number of platelets more commonly associates with severe COVID-19; however, whether the emergence of thrombocytopenia may result in diseases severity or the severity of the disease may decrease platelets, is open to debate.

## Introduction

Since early 2020, almost all people around the world have been tracking the statistics of the novel coronavirus (2019nCoV; named SARS-CoV-2 by the World Health Organization (WHO) in February, 2020 (1)) daily, watching the numbers of infected cases and death tolls increase unceasingly day after day. Life is on hold as death and disease have cast a sinister shadow on people’s lives. It is heartbreaking for the generation in their last decades to die alone and also painful for families losing their loved ones. For the time being (i.e. May 11, 2020), over 4,250,000 infected cases with sorrowful statistics of more than 2850,000 deaths have been recorded all over the world. There are also evidence that adults under the indicated age range with no history of medical conditions may display serious complications and present disease with an unfavorable outcome (2). In other words, coronavirus disease 2019 (abbreviated to COVID-19) is quite eerie, symptomatically, as patients with SARS-CoV-2 infection display a wide range of symptoms – ranging from mild signs to critical care condition requiring specialized management in intensive care units (ICU) (3). 

Although SARS-CoV-2 has a lower mortality rate compared to severe acute respiratory syndrome (SARS)-CoV (10%) (4) and Middle East respiratory syndrome (MERS)-CoV (37%) (5), it has killed a shocking, and of course increasing, number of patients and still continues to infect and take its toll (6). Multiple lines of evidence have attested that viral infections coincide with platelet activation, either through a direct or indirect manner, contributing to increased consumption of these cells (7). It has also been reported that decreased numbers of platelets may serve as a surrogate marker for poor prognosis in a wide range of infectious diseases, including rapidly evolving β-coronaviruses (8), and COVID-19 shall not be considered an exception to this rule (9). Taking advantage of this fact and bearing in mind that the results of several studies reported that low platelet count is associated with increased risk of severe disease (9, 10), it is reasonable to assume that thrombocytopenic COVID-19 patients will experience disease with a higher risk of adverse outcome. To provide a well-conceptualized viewpoint demonstrating the prognostic value of platelet count in SARS-CoV-2 infection, we performed a meta-analysis of pertinent literature to evaluate whether the emergence of thrombocytopenia could discriminate between severe and non-severe cases.

## Methods

The PRISMA guideline was used to pertain to the necessities of performing a systematic review and meta-analysis (11). The search strategy was as follows: the keywords “platelet” OR “thrombocytopenia” AND “COVID-19” OR “coronavirus 2019” OR “2019-nCoV” OR “SARS-CoV-2” were searched in National Library of Medicine Medline/PubMed and Scopus to find articles published between December 30, 2019, and May 9, 2020. The query of ((platelet[Title/Abstract]) OR (thrombocytopenia[Title/Abstract])) AND ((COVID-19[Title/Abstract]) OR (coronavirus 2019[Title/Abstract]) OR (2019-nCoV[Title/Abstract]) OR (SARS-CoV-2[Title/Abstract])) was searched in PubMed in English without any restriction about study type. The results of the initial search were first screened by title and abstract, and then full texts of relevant articles were selected. To strengthen the analysis, the reference list of relevant documents was also scrutinized. All the letters, reviews, editorials, case reports, comments, guidelines, and books were excluded. In addition, all articles that neither presented the information on platelet count nor provided data in the severe and non-severe COVID-19 cases were excluded. The evidence-based librarianship (EBL) Critical Appraisal Checklist was used to assess the eligibility and quality of the studies. Studies with a score higher than 75% were included (12). MS and FH scored the checklist independently, and in the case of disagreement, DB made the final decision.

Meta-analysis was conducted on the mean ± SD platelet counts reported in the included studies to calculate the pooled mean difference in platelet count between severe and non-severe patients. To assess the existence of bias in the included studies, the funnel plot and egger plot along with egger tests were used. In case of bias, the random effect model would be fitted (13). The presence of heterogeneity among the included studies was tested using the Chi-square test, with the significance level of 0.1. After combining results, the level of heterogeneity based on the I^2^ statistic was categorized as follows: low > 25%, moderate > 50, and high > 75% (14). A random-effect model was used to take the study-to-study variability into account. Statistical significance was set to 0.05. The meta set command in STATA was used for meta-analysis. 

## Results

**Figure 1:**
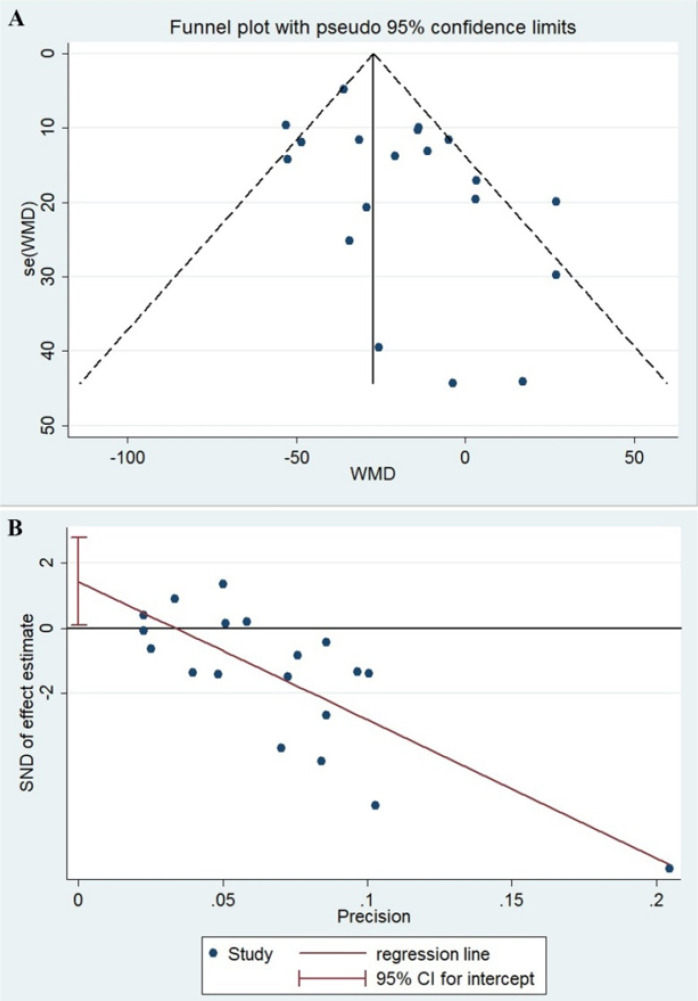
**A)** The funnel plot with pseudo 95% confidence limits for publication bias in the included studies. **B)** The egger plot for publication bias the included studies

**Figure 2:**
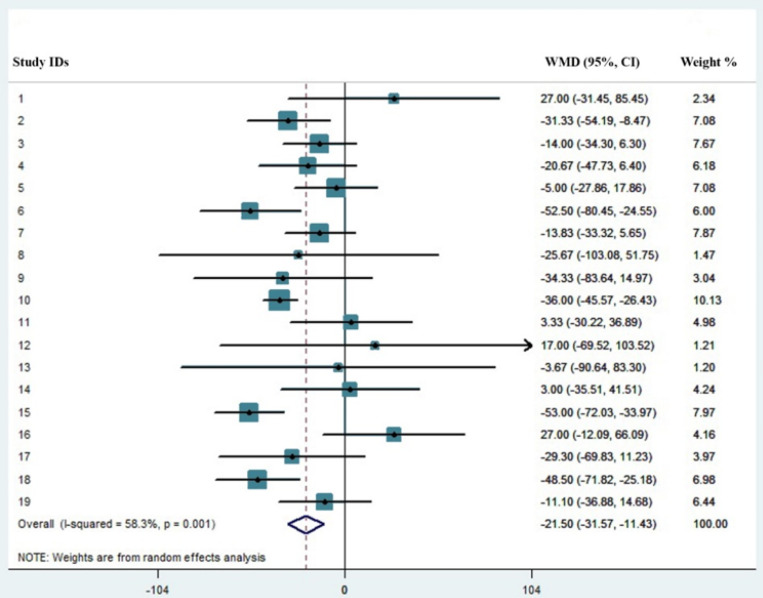
Platelet mean difference count for patients with severe and non-severe COVID-19.

Overall, 4935 articles were identified in our initial search using the indicated criteria and inspecting the reference lists, with a total of 4916 articles being excluded, including 1342 letters, 610 reviews, 418 editorials, 210 case reports, 190 comments, 10 guidelines, and 2 books. The aforementioned articles and also others that either did not fulfill information on the platelet count or provided incomplete information were excluded (2134 articles). The main features of the selected studies including country/city, sample size, and their definition of severe cases are summarized in Table 1. In addition, as represented in this table, demographic characteristics (sample size, age, and sex) of both severe and non-severe COVID-19 patients were collected for all the included studies. On aggregate, we analyzed the results of the platelet count of a total of 3383 patients, 744 (21.9%) of whom were cases with severe disease. The number of cases ranged between 18-1099, whilst the number of severe cases ^___^though with different definitions^___ ^varied between 6-173. The results of platelet count for both severe and non-severe COVID-19 patients were collected from all of the included studies and are shown in Table 2. As represented in Figure 1, the asymmetry in the funnel plot reflects the presence of publication bias, which is also confirmed through the egger plot (Figure 2) and egger test (P=0.04). The *chi*-*square test* for *heterogeneity* was significant (P=0.001), with the I^2^ value of 58%, *implying* medium dissimilarity in the included studies. Then, a random effect model was applied to take into account the heterogeneity and the publication bias. The results revealed that the platelet pooled mean difference (%95 Confidence Interval) was -21.5 [(-31.57, -11.43), P<0.001]. As represented in Figure 2, non-severe cases of COVID-19 had a significantly higher number of platelets compared to patients with severe disease. Although our data show that thrombocytopenic COVID-19 patients experience a more severe disease than SARS-CoV-2-infected cases with normal platelet count, several limitations such as low sample size and uneven description of disease outcome, along with incomplete information on sampling time might have adversely affected our analysis.

**Table 1:** Main features of the selected studies

**Study**	**Country, City**	**Sample size**	**Severity definition**	**Severe patients**			**Non-severe patients**		
				**N, %**	**Age, years**	**Female, %**	**N, %**	**Age, years**	**Female, %**
**Huang et al. (** 24 **)**	China, Wuhan	41	ICU admission	13 (31.7)	49 (41, 61)	2 (15)	28 (68.3)	49 (41, 57.5)	9 (32)
**Cao et al. (** 28 **)**	China, Shanghai	198	ICU admission	19 (9.6)	63.7 (16.8)	2 (10.5)	179 (90.4)	48.6 (15.6)	95 (53.1)
**Zhang et al. (** 29 **)**	China, Wuhan	221	WHO guideline (30)	55 (24.9)	62 (52-74)	20 (36.4)	166 (75.1)	51 (36-64.3)	93 (56)
**Wan et al. (** 31 **)**	China, Chongqing	135	ICU admission; M. Ventilation	40 (29.6)	56 (52-73)	19 (47.5)	95 (70.4)	44 (33-49)	43 (45.3)
**Wang et al. (** 32 **)**	China, Wuhan	138	ICU admission	36 (26.1)	66 (57-78)	14 (38.9)	102 (73.9)	51 (37-62)	49 (48)
**Zhou et al. (** 33 **)**	China, Wuhan	191	Death	54 (28.3)	69 (63, 76)	16 (30)	137 (71.7)	52 (45, 58)	56 (41)
**Gong et al. (** 34 **)**	China, Wuhan/Guangdong	189	NR	28 (14.8)	63.5 (54.5;72)	12 (42.9)	161 (85.2)	45 (33, 62)	89 (55.3)
**Liu et al. (** 35 **)**	China, Chongqing	51	WHO guideline (30)	7 (13.7)	52 (44-60)	3 (42.9)	44 (86.3)	44 (33-49)	16 (36.4)
**Qian et al. (** 36 **)**	China, Zhejiang	91	Respiratory distress/insufficiency	9 (9.9)	66 (54, 80)	NA	82 (90.1)	49 (35.5;56)	NA
**Guan et al.** ^ (^^37^^)^	China, 30 provinces	1099	ICU admission, M. ventilation, Death	173 (15.7)	52 (40, 65)	73 (42.2)	926 (84.3)	45 (34, 57)	386 (41.8)
**Wang et al. (** 38 **)**	China, Wuhan	69	SpO2 < 90	14 (20.2)	70.5 (62, 77)	7 (50)	55 (79.8)	37 (32, 51)	30 (55)
**Fan et al. (** 39 **)**	Singapore	67	ICU admission	9 (13.4)	54 (47-62)	3 (33.3)	58 (86.6)	41 (32-53)	27 (48.6)
**Young et al. (** 26 **)**	Singapore	18	Required supplemental O2	6 (33.3)	56 (47-73)	4 (67)	12 (66.7)	37 (31-56)	4 (42)
**Chen et al. (** 40 **)**	China, Wuhan	21	SpO2 < 93%	11 (52.4)	63.9 (9.6)	1 (9.1)	10 (47.6)	51.4 (13.7)	3 (30)
**Tang et al. (** 41 **)**	China, Wuhan	449	Death	134 (29.8)	68.7 (±11.4)	44 (32.83)	315 (70.2)	63.7 (±12.2)	137 (43.5)
**Yang et al. (** 42 **)**	China, Wuhan	52	Death	32 (61.5)	64·6 (11.2)	11 (34)	20 (38.5)	51·9 (12.9)	6 (30)
**Wei Liu et al. (** 43 **)**	China, Wuhan	78	Admission to ICU, Death	11 (14.1)	66 (51, 70)	4 (36.4)	67 (85.9)	37 (32, 41)	35 (52.2)
**Ruan et al. ** **(** 44 **)**	China, Wuhan	150	Death	68 (45.3)	67 (15, 81)	19 (28)	82 (54.7)	50 (44, 81)	29 (35)
**Wang et al. (** 25 **)**	China, Fuyang/Anhui	125	PaO_2_/FIO_2 _≤100 mmHg	25 (20)	49.4 (±13.64)	9 (36)	100 (80)	39.4 (±14.8)	45 (45)

**N:** Number; **ICU:** Intensive care unit; **WHO:** World health organization; **NR:** Not reported; **M. ventilation:** mechanical ventilation.

**Table 2:** Values of platelet count in severe and non-severe COVID-19 patients

	**Platelet count**	
	**Non-severe**	**Severe**
**Huang et al. (** 24 **)**	149 (131-263)	196 (165-263)
**Cao et al. (** 28 **)**	177 (143-220)	147 (120-179)
**Zhang et al. (** 29 **)**	175 (136-213)	169 (111-202)
**Wan et al. (** 31 **)**	170 (136-234)	147 (118-213)
**Wang et al. (** 32 **)**	165 (125-188)	142 (119-202)
**Zhou et al. (** 33 **)**	220 (168-271)	165.5 (107-229)
**Gong et al. (** 34 **)**	180 (147- 221)	167 (139.5-200)
**Liu et al. (** 35 **)**	192 (139-237)	150 (116-225)
**Qian et al. (** 36 **)**	198 (144-248)	152 (127-208)
**Guan et al.** **(**37**)**	172 (139-212)	137 (99-179)
**Wang et al. (** 38 **)**	172 (138-206)	167 (144-215)
**Fan et al. (** 39 **)**	201 (157-263)	217 (154-301)
**Young et al. (** 26 **)**	159 (128-213)	156 (116-217)
**Chen et al. (** 40 **)**	161 ±44.2	164 ±45.8
**Tang et al. (** 41 **)**	231 ±99	178 ±92
**Yang et al. (** 42 **)**	164 ±74	191 ±63
**Wei Liu et al. (** 43 **)**	173.20 ±55.37	143.90 ±64.81
**Ruan et al. ** **(** 44 **)**	222.1 ±78.0	173.6 ±67.7
**Wang et al. (** 25 **)**	169.5 (142-212)	163.4 ±60.263

Data are presented as mean ± standard deviation or mean (95% confidence interval)**.**

## Discussion

One of the darkest periods in the history of human medicine is recorded in 2020. It is not the dreaded nuclear war, but an outbreak of an insidious member of β-coronaviruses that wages a full-scale war against human health, intimidating the peoples of nations and continents (15). Considering the fact that SARS-CoV-2 infection has brought the national healthcare systems of nearly all countries ^___^even those with highly developed medical facilities^___^ to their knees, battling with this lethal virus seems to be not easy but not impossible either. Regardless of its innate designation (16), the influential role of the laboratory is far beyond the etiological identification of pathogens and it is now practically established that this branch of the medical science is effectively contributing to determining the prognosis of a wide range of human diseases, including COVID-19 (17, 18). Even though the results of a recent study to establish a prediction model for the prognosis of SARS-CoV-2 infection (19) introduced C reactive protein, lactic dehydrogenase, and lymphocyte count as the most valuable laboratory parameters reflecting COVID-19 severity, articles continuously introducing novel biomarkers with the ability to predict disease outcome are published daily. Therefore, considering the results of several recent studies reporting the prognostic value of platelet in patients with COVID-19, it might not be unrealistic to assume that a decreased number of platelets may potentially serve as a simple and readily available biomarker to predict disease severity.

Patients with severe diseases frequently have decreased numbers of platelets, and the emergence of thrombocytopenia is usual in such a situation mainly as a result of unrestricted consumption due to intravascular coagulopathy, often evolving towards disseminated intravascular coagulation (DIC) (20). Notably, the results of our recent meta-analysis revealed that the elevated level of D-dimers may predict the progression of COVID-19 toward an unfavorable prognosis, further highlighting the fact that SARS-CoV-2-induced coagulopathy is responsible for disease severity (21). While the precise explanation for the occurrence of thrombocytopenia in patients with COVID-19 is unknown, direct infection of bone marrow cells by the virus, platelet destruction by the immune system, and platelet aggregation in the lungs, resulting in microthrombi were proposed as the plausible mechanisms underlying decreased platelet counts in those affected with the disease (20, 22). Since platelets are released from fully mature megakaryocytes residing in the lung, structural change or decrease in the pulmonary capillary bed may also provoke unfortunate defragmentation of platelets (23). To provide a clear viewpoint demonstrating the prognostic value of platelet count in this novel infection, we performed a meta-analysis of pertinent literature representing information on the indicated parameter in patients with a clinically validated deﬁnition of severe disease. Notably, the mean and median ages of severely ill patients were more than 50 years old in all the studies, except for Huang et al. (24) and Wang et al. (25) studies. Besides, the percentage of men with severe disease was greater than women, except for the report by Young et al. (26); further highlighting the fact that male sex and old age are amongst significant risk factors for progression of COVID-19 towards an unfavorable outcome. The result of our meta-analysis of 19 studies, totaling 3383 COVID-19 patients with 744 (21.9%) severe cases, revealed that low platelet count is associated with increased risk of severe disease with the pooled mean difference of -21.5 (%95 CI: -31.57, -11.43), and thus proposed that thrombocytopenic COVID-19 patients will experience disease with a higher risk of adverse outcome. In agreement with thee findings, a recent study conducted on 383 patients, 49 (12.8%) of whom died, proposed platelet count as an independent risk factor associated with in-hospital mortality and reported that increment of per 50 × 10^9^/L in platelets was associated with 40% decrease in mortality (hazard ratio: 0.60, 95%CI: 0.43, 0.84) (27). In conclusion, we believe that daily monitoring of the platelet count has a specific clinical significance in this infection and the appearance of thrombocytopenia may mirror the progression of COVID-19 toward an unfavorable outcome. Although the results of this study declared the prognostic significance of platelet count in SARS-CoV-2 infection, several limitations ranging from inadequate studies to fickle study design, either concerning different sampling times or underlying medical conditions, may have adversely affected our analysis. Since in some papers, mean ± SD was only shown in graphs, it was not possible to include them. In addition, several articles did not mention either study population or clear statistical analysis. Asymmetrical form of funnel plot also suggests that there is publication bias, showing overestimation in studies with lower sample size. Finally, since COVID-19 is a recent hot topic and researchers are mostly motivated to publish their papers in local and regional journals as soon as possible, restricting to English-language search was another review-level limitation.

## Conclusion

Daily monitoring of platelet count has specific clinical significance in this infection, and the appearance of thrombocytopenia may mirror the progression of COVID-19 towards an unfavorable outcome. 
